# Focused ultrasound–induced blood–brain barrier opening: A comparative analysis of permeability quantification based on $K^{\text{trans}}$ and PS

**DOI:** 10.1002/mrm.30446

**Published:** 2025-02-18

**Authors:** Denisa Hývlová, Radovan Jiřík, Jiří Vitouš, Ondřej Macíček, Lucie Krátká, Eva Dražanová, Zenon Starčuk

**Affiliations:** ^1^ Institute of Scientific Instruments Czech Academy of Sciences Brno Czechia; ^2^ Faculty of Electrical Engineering and Communication Brno University of Technology Brno Czechia; ^3^ Department of Pharmacology, Faculty of Medicine Masaryk University Brno Czechia

**Keywords:** BBB opening, DCE‐MRI, focused ultrasound, perfusion analysis, simulation

## Abstract

**Purpose:**

Focused ultrasound‐induced blood–brain barrier (BBB) opening is a promising method for neurotherapeutic delivery. The standard for quantifying induced BBB permeability is the $K^{\text{trans}}$ parameter, which reflects both permeability and plasma flow. The influence of plasma flow can be eliminated by estimating the PS parameter. However, this parameter has been largely unexplored in this application. This study aims to compare permeability estimates based on $K^{\text{trans}}$ and PS in focused ultrasound–induced BBB opening experiments.

**Methods:**

We used the extended Tofts model (ETM) and the two‐compartment exchange model (2CXM) to estimate $K^{\text{trans}}$ and PS parameters, respectively. Permeability estimates were compared using simulated concentration curves, simulated DCE‐MRI data, and real datasets. We explored the influence of spatially‐regularized model fitting on the results.

**Results:**

For opened BBB, $K^{\text{trans}}$ was minimally influenced by plasma flow under the tested conditions. However, fitting the ETM often introduced outliers in $K^{\text{trans}}$ estimates in regions with closed BBB. The 2CXM outperformed the ETM at high signal‐to‐noise ratios, but its higher complexity led to lower precision at low signal‐to‐noise ratios. Both these issues were successfully compensated by spatially‐regularized model fitting.

**Conclusion:**

Both $K^{\text{trans}}$ and PS seem to be eligible options for the quantification of BBB opening, and the correct choice depends on the specifics of the acquired DCE‐MRI data. Additionally, spatial regularization has demonstrated its importance in enhancing the accuracy and reproducibility of results for both models.

## 1 INTRODUCTION

The blood–brain barrier (BBB) is a highly selective membrane that separates the bloodstream from the brain tissue, protecting the brain from harmful substances while allowing essential nutrients to enter. Formed by tight junctions between endothelial cells in cerebral blood vessels, the BBB plays a crucial role in maintaining neural environment homeostasis. However, it also poses a significant challenge in treating neurological diseases, as it blocks the delivery of most neurotherapeutics.^1^

Several techniques for drug delivery to the brain have been explored, such as transcranial application, drug lipidization, endogenous transporter modification, and BBB disruption using chemical agents.^2^ A non‐invasive technique called focused ultrasound (FUS)‐induced BBB opening has recently emerged as a promising method to temporarily disrupt the BBB and enhance drug delivery in a targeted brain location. This technique has shown potential in the treatment of brain tumors, Alzheimer's disease, and Parkinson's disease,^1^, ^3^, ^4^ although further research is needed to improve its safety and efficacy in clinical settings.

### 1.1 FUS‐induced BBB opening

FUS‐induced BBB opening involves the use of low‐intensity ultrasound after intravenous administration of microbubbles. Ultrasound waves cause oscillation of these microbubbles, resulting in transient openings in the tight junctions of the BBB. This enables the delivery of therapeutic agents and molecules with diameters up to 65 nm, which would otherwise be unable to cross the BBB.^5^ These openings typically close within hours or days, depending on the ultrasound and microbubble parameters.^5^, ^6^

Safety remains a critical concern in clinical translation, with ultrasound parameters playing a key role. Exceeding safety limits might result in adverse effects such as erythrocyte extravasation, edema, tissue damage, or rapid temperature increase of tissue. The permeability of the BBB is affected particularly by the acoustic pressure, with higher values causing longer‐term and vast BBB opening but also increasing the probability of tissue damage and erythrocyte extravasation.^6^, ^7^

### 1.2 Quantification of BBB permeability

Before applying neural treatment, validation of the BBB opening is necessary. Gadolinium‐based magnetic resonance (MR) contrast agents (CA), typically 1–60 nm in size,^5^ can pass through the open BBB and remain intravascular in areas with an intact BBB, making them ideal for visualizing BBB opening. Dynamic contrast‐enhanced magnetic resonance imaging (DCE‐MRI) is commonly used for this purpose. This technique involves intravenous administration of a gadolinium‐based CA, whose passage through the tissue is captured in a series of MR images. By analyzing the changes in signal intensity over time, several perfusion and permeability parameters might be estimated. First, the signal of each voxel is converted to the CA concentration in tissue c(t) using the measured pre‐contrast scans and a $T_{1}$ mapping method.^8^ The tissue concentration c(t) can be defined using the formula

(1) $$c(t)=(c_{p}*H)(t),$$

which is the convolution of a tissue residue function H(t) and the CA concentration in plasma of the feeding artery $c_{p}(t)$, also known as the arterial input function (AIF). Both concentration curves can be measured, and the unknown in this equation is only H(t). This function can be represented by various pharmacokinetic (PK) models, and its course can be obtained by fitting (1) to the measured concentration curves.^9^ The parameters of the fitted function H(t) are the sought perfusion and permeability parameters, such as plasma flow $F_{p}$, plasma volume $v_{p}$, extravascular extracellular volume $v_{e}$, mean capillary transit time $T_{c}$, extraction fraction E, or permeability–surface product PS, depending on the chosen model.^8^

### 1.3 Pharmacokinetic models and permeability parameters

Based on complexity, PK models can be categorized into the first and the second generations. The first‐generation models are simpler, defined with a limited set of parameters. Considering the permeability quantification, the volume‐transfer constant $K^{\text{trans}}$ describing the influx of CA from the plasma to the extravascular extracellular space can be estimated. This parameter is influenced by the plasma flow $F_{p}$ and permeability–surface area product PS, with two boundary regimes: the flow‐limited case ($\text{PS}\gg F_{p}$) resulting in $K^{\text{trans}}\approx F_{p}$ and the permeability‐limited case ($\text{PS}\ll F_{p}$) resulting in $K^{\text{trans}}\approx \text{PS}$.^9^ The permeability induced by FUS is lower than plasma flow in the brain; therefore, under the assumption of a permeability‐limited case, $K^{\text{trans}}$ might be used for quantification of the BBB opening. However, the difference between PS and $K^{\text{trans}}$ might be non‐negligible, especially for higher permeabilities.

Second‐generation models are more complex, requiring high temporal resolution of the DCE‐MRI sequence, but they separate plasma flow and permeability; thus, they allow quantification of the BBB permeability directly with the PS parameter. Accurate quantification of the BBB permeability is crucial for safety evaluation and planning of the neural treatment; therefore, using the PS as a measure of BBB opening might be more valuable in preclinical and clinical FUS studies.

To the authors' knowledge, only first‐generation models have been applied in FUS‐induced BBB opening studies:

- Patlak,^10^, ^11^
- Tofts,^2^, ^6^, ^12^, ^13^, ^14^, ^15^, ^16^
- and extended Tofts,^7^, ^17^, ^18^, ^19^, ^20^, ^21^, ^22^, ^23^, ^24^, ^25^

all estimating the $K^{\text{trans}}$ parameter as a measure of permeability. The aim of this study is to explore the accuracy limits of the $K^{\text{trans}}$‐based permeability quantification under various conditions of FUS experiments and compare it with the direct estimation of PS using second‐generation models. Preliminary work was published in a conference contribution^26^; here, we present a thorough evaluation and focus also on the improvements in permeability estimation with spatially‐regularized model fitting.

Three PK models were chosen for the comparison: the extended Tofts model (ETM) as the most commonly used first‐generation model and two models from the second generation, the two‐compartment exchange model (2CXM) and its modification, the two‐compartment uptake model (2CUM).^9^ First, we compared the permeability estimates obtained by fitting simulated concentration curves with the selected models. We discussed the validity of the 2CUM and omitted it from the following experiments. Then we conducted the FUS‐induced BBB opening and DCE‐MRI measurement of a total of nine real datasets in three mice. The use of the second‐generation models for the quantification of the BBB permeability with PS requires high temporal resolution (around 1 s per frame). This was provided by the application of a compressed sensing approach, including radial golden‐angle acquisition^27^ and spatio‐temporally regularized image reconstruction.^28^ In addition, the reliability of permeability estimates was improved by spatially‐regularized PK‐model fitting.^29^ To overcome the problem of no ground‐truth information, we simulated synthetic DCE‐MRI datasets based on the real DCE data, using our freely available simulation software PerfSim,^30^ and processed them with the 2CXM and the ETM. The results of permeability estimation with these models were also illustrated on the real datasets.

## 2 METHODS

### 2.1 Experimental

#### 2.1.1 Animal prepararion

Female ICR mice at the age of 15 weeks weighing 30 g were purchased from Charles River, Germany. The experiment was conducted in three animals. All procedures were performed under EU Directive no. 2010/63/EU and approved by the Animal Care Committee of the Czech Academy of Sciences in compliance with Czech Animal Protection Act No. 246/1992.

Each mouse was anesthetized with a mixture of 1.5–2% isoflurane and 800 mL/min of oxygen‐enriched air. For the administration of ultrasound microbubbles and gadolinium CA, the caudal vein was cannulated. After cannulation, the scalp fur was depilated to minimize acoustic impedance mismatch during the sonication, and the animal was transferred to the water‐heated animal bed in a MR scanner room. The body temperature and respiratory curve were monitored during the whole experiment.

#### 2.1.2 FUS

Vevo® MicroMarker (FUJIFILM VisualSonics, Canada) ultrasound microbubbles were administered intravenously (50 μL followed by 150 μL of saline), and the mouse was sonicated using MRI‐guided FUS for small‐bore preclinical imaging systems (RK‐300, FUS Instruments, Toronto, Canada) with a piezoelectric transducer of a resonant frequency = 1.447 MHz, diameter = 2.5 cm, and focal length = 2 cm. The sonication parameters were set to 0.8 MPa pressure amplitude, 10 ms burst duration, 1000 bursts per ms, and 100 bursts in total.

#### 2.1.3 DCE‐MRI

Upon BBB opening, we acquired three DCE‐MRI sequences—right after sonication (approx. 15 minutes), after 4 h, and after 23 h—to observe the dynamics of the BBB closing. The imaging was performed on a 9.4T Bruker BioSpec USR 94/30 (Bruker BioSpin, Ettlingen, Germany) scanner with a surface mouse‐brain four‐channel array coil. For $T_{1}$ mapping, a radial golden‐angle stack‐of‐stars (GA SOS)^27^ inversion recovery Look–Locker (IRLL) pre‐contrast sequence^31^ was acquired with τ = 8.2 ms, $t_{d}$ = 12.1 ms, $t_{r}$ = 8.2 ms, FA = 3 °, TE = 1.6762 ms, 1500 projections per inversion, and 3200 projections in total. The DCE imaging was performed with an RF‐spoiled 3D FLASH sequence with a GA SOS readout^31^ using a bolus of 0.2 mmol/kg Gadovist® (Bayer AG, Germany) CA was administered intravenously 45 s after the start of scanning using a linear infusion pump (Harvard Apparatus) with an injection speed of 1 mL/min. The parameters of the sequence were TR = 8 ms, TE = 1.445 ms, FA = 15 °, 38400 projections, and total acquisition time = 256 s. The FOV of both sequences was 35 × 35 × 12 mm, the matrix size of the reconstructed images was 128 × 128 × 10, and the spatial resolution 0.273 × 0.273 × 1.2 mm per voxel.

#### 2.1.4 Data processing

Acquired data were reconstructed slice‐by‐slice using the BART toolbox^28^ with total‐generalized‐variation regularization in both the spatial and the temporal domains. The temporal sampling period of the DCE sequence was set to 1.2 s.

Perfusion analysis was performed using the PerfLab software^32^ designed for quantitative DCE‐MRI analysis. First, the image sequence was converted to CA concentration using the IRLL $T_{1}$ mapping.^31^ We measured the AIF manually from a large brain artery. The data were fitted with the 2CXM and ETM (using a trust‐region‐reflective algorithm). Standard voxelwise and spatially‐regularized PK‐model fitting was used. Spatially‐regularized model fitting was done in a similar way as in,^29^ with an iteratively regularized Gauss‐Newton method implemented.^33^

The model performance was quantified with the Akaike information criterion (AIC), a statistical metric gauging the goodness of fit considering the number of model parameters.^34^ For the least‐squares regression task, the AIC can be calculated as

(2) $$\text{AIC}=N\cdot \ln\left(\frac{\text{SSD}}{N}\right)+2(P+1),$$

where SSD is the sum of square differences, N is the number of time samples, and P is the number of model parameters.^35^, ^36^

We estimated the voxelwise AICs for the voxelwise‐fitted ETM and 2CXM, separately in regions with opened and closed BBB, which were manually selected in all nine image sequences (one slice selected per sequence). The model performance was then evaluated as a difference between model AICs, $\Delta_{\text{AIC}}={\text{AIC}}_{\text{ETM}}-{\text{AIC}}_{\text{2CXM}}$. To cope with high variability in single‐voxel AICs, the median and the 25th and 75th percentiles across the two regions and all nine recordings were reported.^37^

### 2.2 Simulations

#### 2.2.1 Simulation of the concentration curves

To assess the accuracy and precision of the permeability estimates without the influence of the imaging process, we simulated concentration curves and fitted them with the chosen PK models. For this purpose, the PerfSim^30^ and PerfLab^32^ functions were utilized to generate and process the data.

The concentration curves were simulated for FUS‐induced permeability varying in the range of PS = [0.0, 0.1] mL/min/mL with a step of 0.01 mL/min/mL, that is, from no permeability to values on the verge of tissue damage. The remaining parameters were set to the values expected in a healthy brain, that is, $F_{p}$ = 0.8 mL/min/mL, $v_{e}$ = 0.1 mL/mL and $T_{c}$ = 0.01 min.^38^, ^39^ The IRF functions were simulated using the 2CXM, that is, the most realistic PK model of the tested ones. The three‐gamma‐variate function (3GVF) AIF,^40^, ^41^ derived for small animals, was generated and convolved with the function H(t) (Equation 1) so that the concentration curves were obtained. The curves were generated with the total acquisition time of 256 s and subsampled to $T_{s}$ = 1.2 s to match the parameters of the real and synthetic DCE‐MRI data.

Apart from the noise‐free concentration curves, two sets of concentration curves with Gaussian noise were created with standard deviations (SD) of the noise of 0.001 and 0.005 mmol/L, and 20 different realizations of the noise. The curves were fitted with the chosen PK models—ETM, 2CXM, and 2CUM.

The 2CUM considers the backflux of the CA negligible compared to the influx. This is valid when the permeability is low and the total acquisition length is short. In practice, the acquisition length should be shorter than^9^

(3) $$T_{e}<\frac{v_{e}}{\text{PS}}.$$

Therefore, the concentration signals were shortened for the fitting of the 2CUM to 100 s so that the highest permeability (0.06 mL/min/mL) assigned to the virtual PerfSim phantom described in the following section met this condition.

#### 2.2.2 PerfSim: DCE data simulator

The simulator PerfSim was used to generate the synthetic‐phantom DCE‐MRI data.^30^ The input for the simulation is a virtual phantom composed of segmented tissue regions, where each tissue is assigned a corresponding set of perfusion parameters. The simulator generates the concentration curves using a predefined PK model and AIF for each region of interest and converts them, based on FLASH acquisition, to MR signal intensity. A synthetic image sequence with high temporal and spatial resolution is then created from the signal curves and the virtual phantom and weighted by input coil sensitivities. MR echo signals are generated by transforming the synthetic image sequence into k‐space. The simulator allows Cartesian, radial, and rosette k‐space sampling. Gaussian noise may be added to the echo signals. The software implements simulation of multi‐FA, multi‐TR, and IRLL pre‐contrast sequences.

A virtual phantom complying with the FUS experiment was designed, representing an axial slice of a rat's head with the BBB opened in a targeted area. Several regions representing the opened BBB were added to the virtual phantom and assigned corresponding perfusion parameters. The permeability in the area was defined by PS and set to four levels, PS = {0.2, 0.4, 0.5, 0.6} mL/min/mL, with PS decreasing with the distance from the focus. The values are based on perfusion analysis of the DCE‐MRI sequences acquired right after BBB opening. The $F_{p},v_{e}$ and $T_{c}$ parameters were considered unaffected by the FUS. The perfusion parameters of the remaining tissues were assigned based on analysis of real DCE data and literature, similarly to.^29^

**FIGURE 1:**
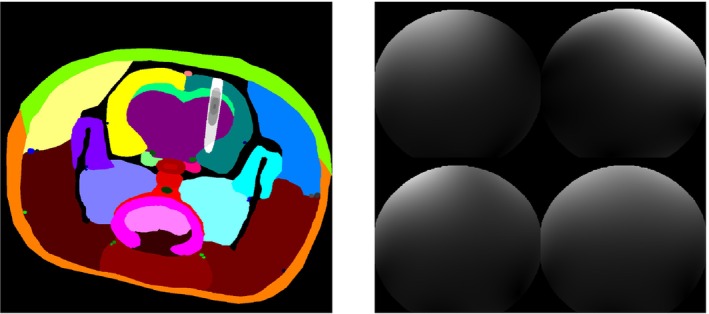
Input data of simulations—virtual phantom and coil sensitivity.

The input data are shown in Figure 1—the virtual phantom (left) with regions representing the opened BBB depicted in shades of gray, and the sensitivity of a surface four‐channel array coil (right) estimated from a phantom measurement.

#### 2.2.3 Simulation of the DCE dataset

The synthetic DCE dataset was simulated with the acquisition parameters similar to the animal experiment described in Section 2.1.3. Three datasets were generated:2D, 2D with noise, and 3D with noise (with 10 slices as the real acquisition). 3D simulation is greatly facilitated by the SOS acquisition with the same radial sample positions (given by the same angle of the spokes) for each of the equidistantly distributed slice‐encoded ($k_{s}$) scans. Therefore, the acquired k‐space data can be separated into single‐slice data using the 1D Fourier transform in the $k_{s}$ direction. That allows the simulation of a single‐slice part of the 3D k‐space dataset based only on a single‐slice phantom.

We set the SD of Gaussian noise added to the echo signals as 0.022 to achieve a similar level of noise as in the real datasets. The IRLL pre‐contrast sequence^31^ was simulated with the parameters of the real acquisition (Section 2.1.3).

The image sequences were reconstructed from the synthetic k‐space data using the BART reconstruction toolbox^28^ with total‐variation regularization in both the spatial and the temporal domains. The temporal sampling of the DCE sequence was set to 1.2 s, corresponding to the sampling of the real acquisition.

The reconstructed data were processed using the PerfLab software. The 3GVF AIF, which was used for the simulation of the dynamic sequence, was selected to deconvolve the signals. Based on previous analysis of the concentration curves, only the ETM and the 2CXM were used for the perfusion analysis, fitted both voxelwise and with spatial regularization. The $\Delta_{\text{AIC}}$ was calculated, as described in Section 2.1.4, separately for the three simulated datasets.

## 3 RESULTS

### 3.1 Concentration‐curve analysis

**FIGURE 2:**
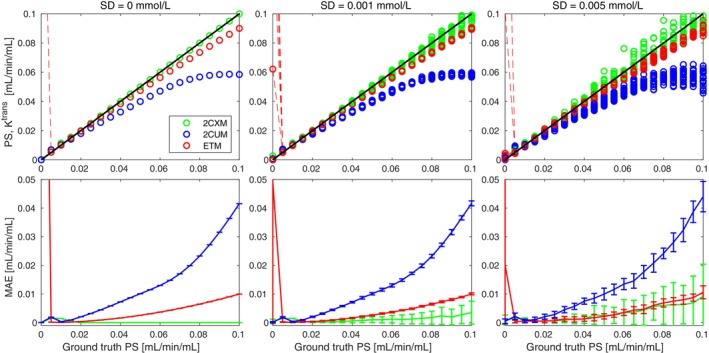
Permeability estimates from the 2CXM (green), 2CUM (blue), and ETM (red) compared with the ground‐truth PS (black) for varying permeability (top row) and mean absolute error of the estimates (bottom row). Columns correspond to SD = 0, 0.001, and 0.005 mmol/L of the added noise, respectively.

The results of the concentration‐curve fitting with the selected models are shown in Figure 2. All three models were able to estimate low permeabilities correctly. The error of the 2CUM increased rapidly with the increasing PS. This behavior was expected for the PS closer to 0.1 mL/min/mL, as the backflux of the CA is no longer negligible for higher PS. Based on Equation (3), the model should fit correctly for PS lower than 0.06 mL/min/mL, but the mean absolute error (MAE) in this range was much higher compared to the other PK models; therefore, for subsequent analysis, only the 2CXM was chosen for the estimation of the PS parameter.

Overall, the 2CXM outperformed the ETM across simulated permeabilities, with the ETM clearly underestimating the BBB permeability for higher PS. In the presence of noise, the estimation of PS becomes less precise than the $K^{\text{trans}}$ estimate, with increasing imprecision for higher permeabilities and noise level. That is due to the uncertainty of optimization for the 2CXM, which has 4 parameters to fit as opposed to the ETM with 3 parameters. For the highest level of noise, the MAE of the 2CXM and the ETM becomes comparable for the opened BBB because of this phenomenon.

**FIGURE 3:**
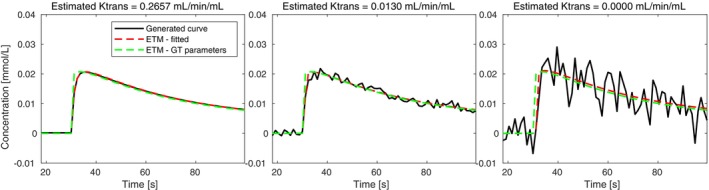
Simulated concentration curves with $T_{s}$ = 1.2 s, $K^{\text{trans}}$ = 0 mL/min/mL, and different noise levels (black), fitted ETM curve (red), and ETM curve generated with ground‐truth parameters (green). Columns correspond to SD = 0, 0.001, and 0.005 mmol/L of the added noise, respectively, as in the previous analysis. The signals were cropped in time to better observe the differences.

Furthermore, the ETM showed instability of the zero‐permeability estimate, often finding a high‐$K^{\text{trans}}$ curve as the global optimum (first sample; connected to the next sample by a red dashed line for the visualization of the estimates outside of the y‐axis range). In the presence of noise, this phenomenon becomes less deterministic, and the low‐$K^{\text{trans}}$ curve sometimes becomes global optimum, depending on the noise realization. The optimization problem is demonstrated in detail in Figure 3, where the time course of the simulated concentration curves (with noise levels as in Figure 2) is shown, altogether with the fitted ETM curves (red) and the curves generated with ground‐truth parameters (green). As can be observed, the most important part of the signal curve for the correct estimation of parameters is the arrival of CA into the tissue. For a densely sampled concentration curve without the presence of noise (left), the ETM curve with correct parameters clearly is not the global optimum of the fitting procedure. This indicates that the ETM does not model the zero‐PS case appropriately (explained theoretically below in Discussion). With the presence of noise, the difference between the global and the local optimum is decreasing, possibly resulting in correct parameter estimation, depending on the specific noise realization.

**FIGURE 4:**
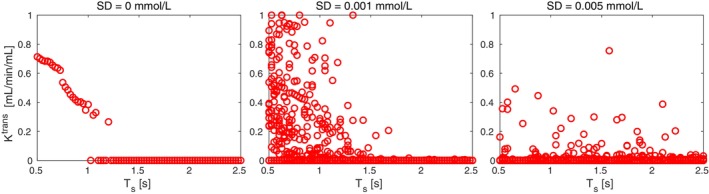
Permeability estimates from the ETM for PS = 0 mL/min/mL for varying sampling period $T_{s}$ and increasing noise levels.

Reduced sampling rate might possibly have a similar effect in the zero‐PS region as the noise, resulting in insufficient capture of the first CA bolus pass. To further explore this behavior, we simulated the concentration curves with PS = 0 mL/min/mL, different noise levels, and with the sampling period varying in the range of 0.5 to 2.5 s, and we fitted them with the ETM (Figure 4). Under the ideal conditions (no noise), the ETM with high $K^{\text{trans}}$ (i.e., incorrect) is the global optimum for the fitting procedure up to $T_{s}$ = 1 s (left graph). For higher $T_{s}$, the curve with $K^{\text{trans}}$ = 0 mL/min/mL (i.e., the correct permeability estimate) becomes the global optimum. In the presence of noise, the results of optimization are highly dependent on the noise level and its realization. Particularly, for high noise levels approaching real‐world conditions (right graph), the correct $K^{\text{trans}}$ = 0 mL/min/mL value predominantly emerged as the global optimum across all examined sampling periods, with occasional incorrect estimations for specific noise realizations. The results for the lower noise level (middle graph) are exhibiting characteristics of both no‐noise and high‐noise curve fitting, providing incorrect $K^{\text{trans}}$ estimates for lower $T_{s}$ more frequently.

### 3.2 Simulated‐dataset analysis

**FIGURE 5:**
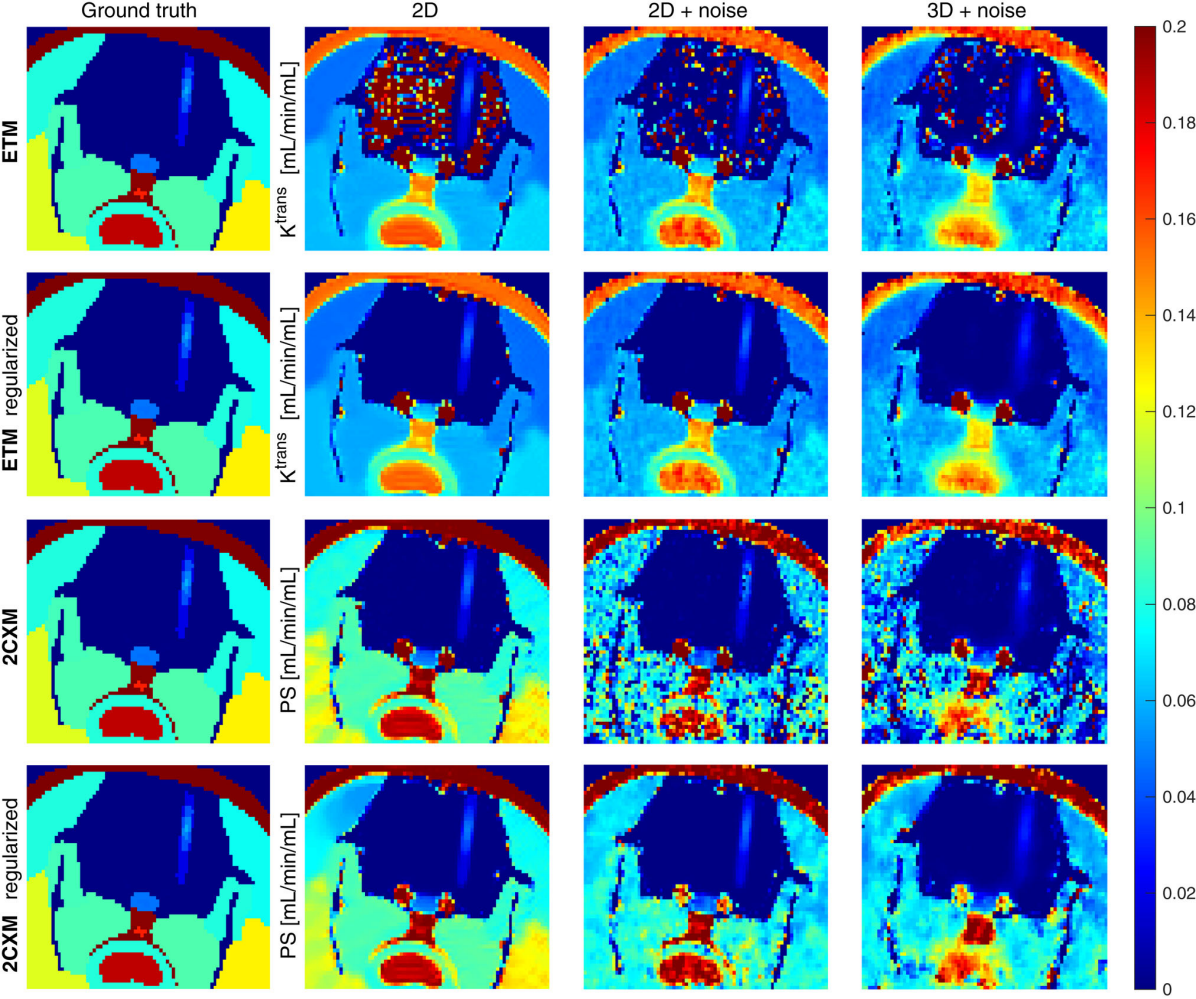
Permeability estimates from the voxelwise and regularized ETM and 2CXM on 2D noise‐free data, 2D noisy data, and 3D noisy data, compared with the ground‐truth PS (mL/min/mL).

The results of the synthetic‐dataset perfusion analysis using both models were compared with the ground‐truth PS, shown in a close‐up view of the virtual phantom in Figure 5. The voxelwise $K^{\text{trans}}$ estimate from the ETM (first row) was overall more precise and less affected by noise than the voxelwise PS estimate (third row); however, unstable for the intact BBB. The presence of noise resulted in more frequent estimation of the correct zero permeability for the intact BBB, confirming the model's behavior shown previously in Figure 2. The uncertainty of the voxelwise 2CXM estimate when the noise is present manifested similarly as in the case of concentration‐curve fitting (Figure 2). Imprecisions were observable for non‐zero permeabilities, primarily in highly permeable tissues surrounding the brain, but also in the region with opened BBB.

Spatial regularization improved the performance of both models. The spatially‐regularized ETM (second row) converged to the correct zero‐permeability estimates in the intact brain in all datasets. The PS maps from the spatially‐regularized 2CXM (fourth row) were more spatially consistent, with estimates closer to the ground truth in the FUS lesion. The same effect occurred also in the non‐brain tissues (here the ETM fails to model the concentration curves correctly because the permeability‐limited condition of the ETM does not hold). We quantified the spatial coherency of the permeability estimates in the brain with the Pearson correlation coefficient, which confirmed the visual improvement (Table S1).

Since the ground‐truth map of PS was known, the MAE and SD of the estimation error could be determined. The values are summarized in Table 1 for the voxelwise and spatially‐regularized ETM and 2CXM in the regions with opened BBB and closed BBB. The SD was reported as a relative value due to its small values.

**TABLE 1:** Mean absolute error (mL/min/mL) of the BBB permeability estimates with respect to the ground‐truth PS map and standard deviation (%) of the estimation error.

BBB	Opened			
Model	ETM	ETM 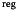	2CXM	2CXM 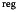
2D	0.0091 ± 0.71 %	0.0092 ± 0.72 %	0.0090 ± 0.71 %	0.0087 ± 0.73 %
2D‐noise	0.0085 ± 0.74 %	0.0087 ± 0.75 %	0.0130 ± 1.90 %	0.0085 ± 0.73 %
3D‐noise	0.0126 ± 0.72 %	0.0125 ± 0.72 %	0.0110 ± 0.58 %	0.0124 ± 0.73 %

BBB	Closed			
Model	ETM	ETM 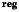	2CXM	2CXM 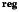
2D	0.3600 ± 42.82 %	0.0019 ± 1.36 %	0.0041 ± 2.97 %	0.0017 ± 1.10 %
2D‐noise	0.0773 ± 21.63 %	0.0019 ± 1.29 %	0.0133 ± 30.23 %	0.0017 ± 0.92 %
3D‐noise	0.0728 ± 20.11 %	0.0035 ± 1.50 %	0.0060 ± 1.61 %	0.0034 ± 1.09 %

For the opened BBB, the performance of all models was comparable, except for the voxelwise 2CXM in the noisy 2D dataset, where the estimation error was higher due to outlier voxels (Figure 5, row 3, column 3). In contrast, the error was slightly lower in the noisy 3D dataset. The same trend of results was obtained when comparing the models in terms of maximum and mean permeabilities estimated within a small region around the sonication focus (Tables S2 and S3).

For the closed BBB, the ETM showed significant performance improvement when regularization was applied, as the voxelwise ETM estimates were predominantly outliers. Spatial regularization also enhanced the performance of the 2CXM. When comparing the regularized models, both achieved low MAEs and SDs, with the 2CXM demonstrating slightly better overall performance.

### 3.3 Real‐dataset analysis

**FIGURE 6:**
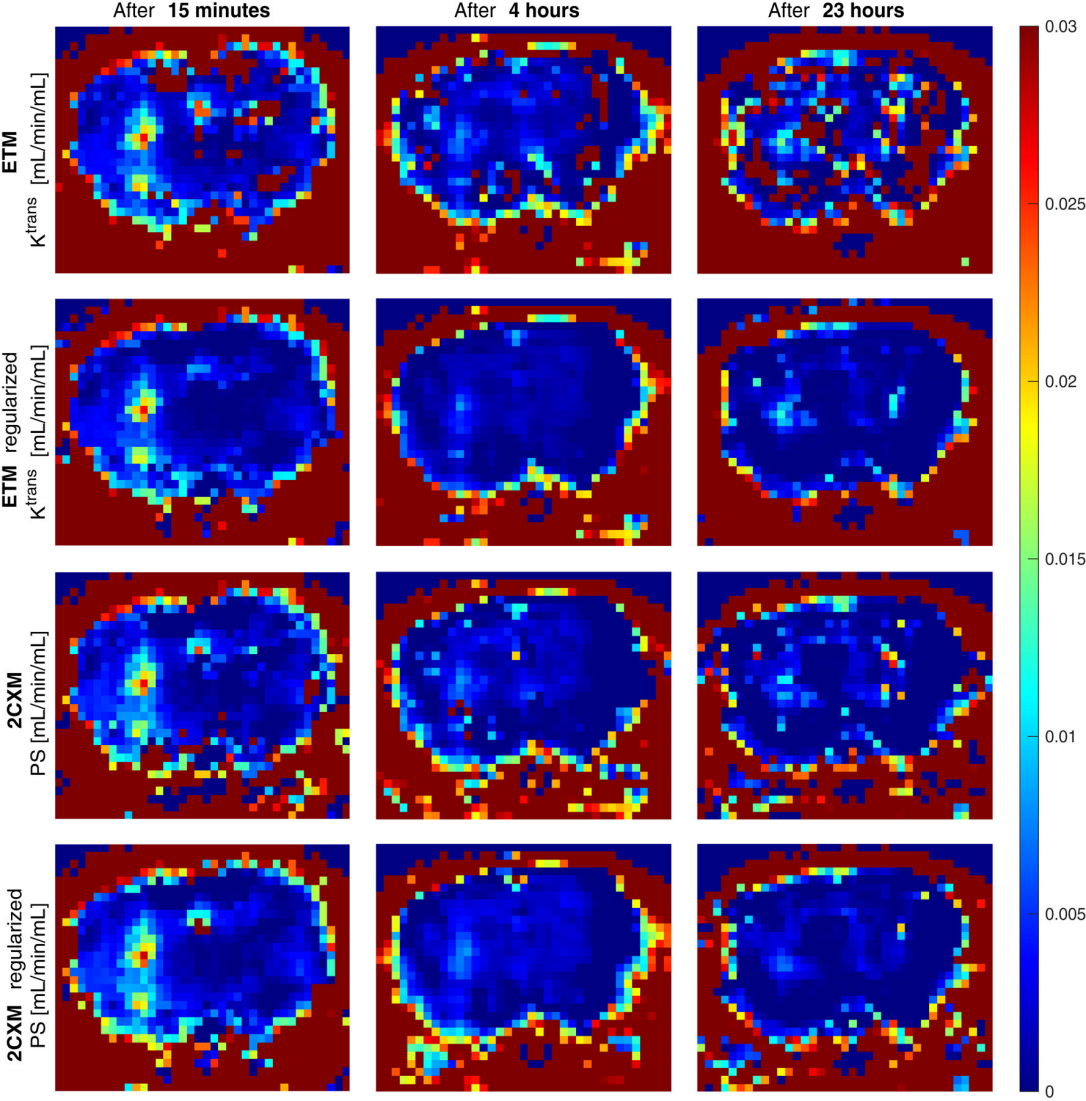
Permeability estimates from the voxelwise and regularized ETM and 2CXM on real mouse data for the three measurements in time after sonication.

Example estimates of the BBB permeability acquired with voxelwise and spatially‐regularized ETM and 2CXM in one animal are shown in a close‐up view of the brain in Figure 6. The columns correspond to the three acquisitions in time. Right after the sonication (left column), there was an apparent region of the BBB opening. After 4 hours (middle column), the BBB was still slightly open, although the permeability had rapidly decreased. The last DCE‐MRI after 23 hours (right column) showed further decrease in permeability, though at a much slower rate.

The levels of BBB opening were estimated similarly across all approaches. The non‐regularized ETM resulted in excessive outliers in the normal brain tissue, as in simulated datasets. Spatial regularization improved the estimates for both models, reducing outliers in the intact brain tissue and enhancing spatial coherence in the sonicated area. This improvement was particularly notable in later measurements at lower levels of BBB opening. The dynamics of BBB closure were best observed in the regularized 2CXM maps, which provided the most visually clear representation. In contrast, the regularized ETM estimated higher permeability in the third measurement compared to the second.

### 3.4 Model‐performance analysis

The results of the model performance analysis are shown in Table 2. Negative values of the $\Delta_{\text{AIC}}$ indicate better ETM performance; positive values indicate better 2CXM performance. For the idealistic noise‐free 2D dataset (first row), the 2CXM fitted the data considerably better when the BBB was closed. For the opened BBB, the median $\Delta_{\text{AIC}}$ indicates slightly better ETM performance; however, the $\Delta_{\text{AIC}}$ value distribution was highly uneven, as shown by the 25–75th percentiles, with the 2CXM performing clearly better than the ETM in some cases. For the noisy 2D and 3D simulated datasets, the ETM performed slightly better for the opened BBB across the entire 25–75th percentile. For a closed BBB, the model performance was more case‐dependent, with a slightly skewed distribution favoring the ETM.

**TABLE 2:** AIC difference of the ETM and 2CXM in simulated and real data. Negative values indicate better performance of the ETM than the 2CXM, and vice versa.

	BBB	
Data	Opened	Closed
Sim.–2D	−2.02 [−2.03, 82.29]	105.32 [19.86, 234.48]
Sim.–2D‐noise	−2.00 [−2.86, −1.22]	−1.16 [−2.09, 2.05]
Sim.–3D‐noise	−2.08 [−2.71, −1.91]	−1.41 [−2.10, 2.68]
Real–all	−2.62 [−15.19, −0.97]	−2.00 [−2.80, 2.70]

In real data, closed‐BBB results were similar to those of noisy simulated data. For the opened BBB, the $\Delta_{\text{AIC}}$ showed a slightly better performance of the ETM compared to the results in the simulated data. This might be caused by a wider range of induced permeabilities compared to the virtual‐phantom data (maximum PS value in the sonicated region ranges from 0.008 to 0.1 mL/min/mL in the real datasets), with the ETM fitting the concentration curves better than the 2CXM for some untested permeabilities.

## 4 DISCUSSION

The experiments have shown that, under the tested conditions, both the ETM and 2CXM can be used to quantify the BBB permeability in the FUS lesions, that is, in regions with non‐zero permeability within the tested range of up to 0.1 mL/min/mL. Here, the performance of both models was comparable.

In the regions of intact BBB, the ETM tends to provide false high $K^{\text{trans}}$ values. This misestimation becomes less frequent with increasing noise level and temporal sampling interval. This effect is in line with the pharmacokinetics theory. The tissue residue function of the ETM is defined as

(4) $$H_{ETM}(t)=v_{p}\delta (t)+K^{\text{trans}}e^{-\frac{K^{\text{trans}}}{v_{e}}t}.$$

For intact BBB, the brain tissue is modeled as a single (intravascular) compartment with a mono‐exponential residue function, as often used in dynamic susceptibility contrast (DSC)‐MRI:

(5) $$H_{DSC}(t)=F_{p}e^{-\frac{F_{p}}{v_{p}}t}.$$

Thus, for intact BBB, under ideal conditions (no noise, high temporal resolution), the ETM fits the concentration curve accurately when $K^{\text{trans}}=F_{p}$ and $v_{p}$ = 0 mL/mL. This corresponds to the cases of false non‐zero $K^{\text{trans}}$ values estimated as the global optimum of the fitting problem. This issue also arises for small non‐zero BBB permeabilities (up to ∼1e‐4 mL/min/mL), as we have shown in our previous conference paper.^26^

In other cases, wherever the ETM provides correct zero $K^{\text{trans}}$, the ETM degenerates to the tissue residue function $H(t)=v_{p}\delta (t)$. This solution is, however, only a local optimum of the fitting problem under ideal conditions. For a higher noise level and temporal sampling interval (as in real data), this local optimum can become a global optimum, leading to the estimation of the correct low $K^{\text{trans}}$. This means that for the intact BBB, under ideal conditions, $K^{\text{trans}}$ estimated by the ETM quantifies the brain permeability less accurately than PS estimated by the 2CXM. However, for real data distorted by noise, PS and $K^{\text{trans}}$ estimated by the 2CXM and ETM are mostly comparable due to the positive influence of noise on the ETM estimates of $K^{\text{trans}}$.

The AIC‐based analysis of model performance favored the ETM, mostly thanks to the lower number of parameters. The common difference in AICs was by 2, originating from the second term 2(P+1) in Equation (2). This means that both models fit the concentration curves equally well; however, the 2CXM is penalized for the additional parameter, which might cause higher variation in the parameter estimates. It is important to note that AIC only reflects the fitting error and the number of model parameters. It does not reflect the parameter estimation error, thus not the $K^{\text{trans}}$ outliers produced by the ETM in closed‐BBB regions. This suggests that the AIC may not be a suitable criterion for model selection in this case.

The proposed concept of spatial regularization has improved both PK models' fitting in the sense of better spatial consistency and suppressed outliers. Spatially‐regularized model fitting compensated for the higher number of 2CXM parameters, which had caused lower precision of the estimates. For the ETM, spatial regularization has also minimized the problem of false high‐permeability estimates in areas with an intact BBB. This might be due to the regularization algorithm penalizing abrupt spatial transitions to the model of Equation (5). However, the results were sensitive to the parameters of model fitting, particularly the user‐specified number of precontrast images, as these parameters affect convergence to different fitting optima (as demonstrated in Figure 3).

Although the simple ETM seems to perform well overall, a second‐generation PK model might offer several advantages. First, advanced models, described with more parameters, provide additional insights with parameters that cannot be estimated with the ETM, such as plasma flow or extraction fraction; see the 2CXM's plasma‐flow maps shown in Figures S1 and S2 and the significant improvement achieved by spatial regularization. Flow maps might be of particular use in applications of FUS in stroke,^42^ as flow maps are used for identification of penumbra. Furthermore, permeability maps from these models can be used for the evaluation of all tissue types where the permeability‐limited regime does not hold, such as penumbra in stroke. This makes second‐generation models more suitable for studies involving multiple tissues or parameters. Additionally, in studies requiring precise estimation of low permeability, such as those examining BBB dynamics, the ETM may not provide reliable results compared to a second‐generation model. In such studies, the 2CUM may also be valid (if the permeability is low enough, see Figure 2 and Equation (3)), and, thanks to the lower number of parameters, offers a more stable optimization process than the 2CXM.

In this work, we used our DCE‐MRI simulator to quantify errors relative to ground truth permeability. However, it is important to acknowledge the limitations inherent in such simulations. For instance, the representation of tissues as constant‐parameter regions in the virtual phantom does not accurately reflect the complexities observed *in vivo*. Additionally, we did not model the inhomogeneities of the main $B_{0}$ field, partially arising from variations in magnetic susceptibility between tissues, and the related susceptibility artifacts, nor the flow artifacts, resulting from dynamic changes in blood flow. The absence of these factors may reduce the fidelity of the simulated results compared to real‐world acquisitions. Despite that, the PerfSim simulator remains a powerful tool for preclinical research, presenting a reasonable compromise between the simulation realism and the computational complexity.

## 5 CONCLUSIONS

We compared the quantification of FUS‐induced BBB permeability using the PS parameter with the standard $K^{\text{trans}}$ estimate, which includes the influence of plasma flow. Two pharmacokinetic models were particularly tested: the commonly used ETM for $K^{\text{trans}}$ estimation and the more complex 2CXM for PS estimation.

Under the tested conditions, $K^{\text{trans}}$ was minimally influenced by plasma flow. However, the ETM frequently introduced outliers in $K^{\text{trans}}$ estimates in areas with an intact BBB. Conversely, the 2CXM outperformed the ETM at high signal‐to‐noise ratios; however, it suffered from reduced precision at lower signal‐to‐noise levels. We successfully addressed these issues by applying spatial regularization during model fitting. Both regularized models produced comparable results, though permeability maps from real data suggest possible superiority of the 2CXM, particularly for lower permeabilities.

In conclusion, both $K^{\text{trans}}$ and PS seem to be eligible options for the quantification of BBB opening, and the correct choice depends on the specifics of the dataset.

## CONFLICT OF INTEREST STATEMENT

The authors declare no potential conflict of interests.

## Supporting information




**Data S1.** Supporting Information.

## Data Availability

Synthetic and real DCE‐MRI data are openly available at https://doi.org/10.5281/zenodo.13683103.^43^ The simulation software PerfSim is freely available at our GitHub page https://github.com/NMRISIBrno/PerfSim.git .^30^
