# Supplementary material for: Focused ultrasound–induced blood–brain barrier opening: A comparative analysis of permeability quantification based on Ktrans and PS
Source: Magn Reson Med. 2025 Feb 18;93(6):2610–22. doi: 10.1002/mrm.30446 (PMC11971499; doi:10.1002/mrm.30446)
Supplement: Supplementary file 1 — Data S1. Supporting Information. [file MRM-93-2610-s001.pdf]

Focused Ultrasound–Induced Blood–Brain Barrier Opening: A Comparative Analysis of Permeability Quantification Based on Ktrans and PS

Denisa Hývlová[1,2*], Radovan Jiřík[1], Jiří Vitouš[1,2], Ondřej Macíček[1], Lucie Krátká[1], Eva Dražanová[1,3], Zenon Starčuk Jr.[1]

[1] Institute of Scientific Instruments, Czech Academy of Sciences, Brno, Czechia
[2] Faculty of Electrical Engineering and Communication, Brno University of Technology, Brno, Czechia
[3] Department of Pharmacology, Faculty of Medicine, Masaryk University, Brno, Czechia

* Corresponding author:
Denisa Hývlová, Institute of Scientific Instruments, Czech Academy of Sciences, Brno, Czechia, email: hyvlova@isibrno.cz

Supplementary Data

Table S1: Pearson correlation coefficient (PCC) estimated in the brain for the simulated 2D noise-free data, 2D noisy data and 3D noisy data.

**PCC**	2D	2D + noise	3D + noise
ETM	0.3095	-0.1824	-0.0544
ETM regularized	0.4161	0.4332	0.2731
2CXM	0.2150	0.0156	0.3095
2CXM regularized	0.5040	0.5585	0.3594

Table S2: Maximum ground-truth and estimated open-BBB permeability from the voxelwise and regularized ETM and 2CXM on 2D noise-free data, 2D noisy data and 3D noisy data. Maximum value was taken from a small region around the focus of sonication.

**Max permeability** [mL/min/mL]	Ground truth	2D	2D + noise	3D + noise
ETM	0.06	0.0472	0.0499	0.0328
ETM regularized	0.06	0.0470	0.0493	0.0331
2CXM	0.06	0.0472	0.0784	0.0452
2CXM regularized	0.06	0.0489	0.0464	0.0310

Table S3: Mean ground-truth and estimated open-BBB permeability from the voxelwise and regularized ETM and 2CXM on 2D noise-free data, 2D noisy data and 3D noisy data. Mean value was taken from a small region around the focus of sonication.

**Mean permeability** [mL/min/mL]	Ground truth	2D	2D + noise	3D + noise
ETM	0.0517	0.0405	0.0414	0.0297
ETM regularized	0.0517	0.0404	0.0412	0.0298
2CXM	0.0517	0.0405	0.0353	0.0354
2CXM regularized	0.0517	0.0418	0.0404	0.0287

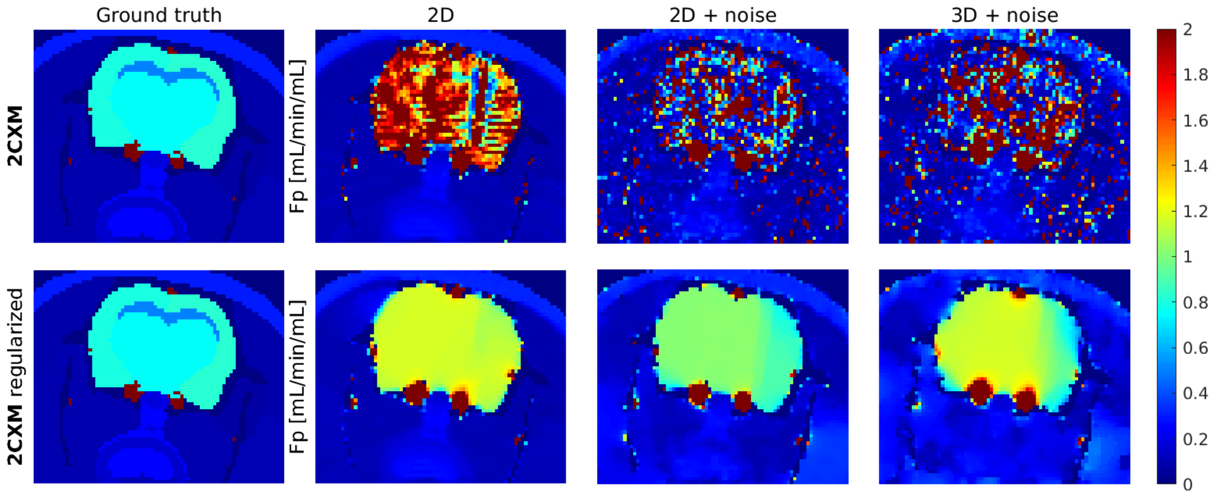

Figure S1: Plasma flow estimates from the voxelwise and regularized 2CXM on 2D noise-free data, 2D noisy data and 3D noisy data, compared with ground-truth Fp [mL/min/mL].

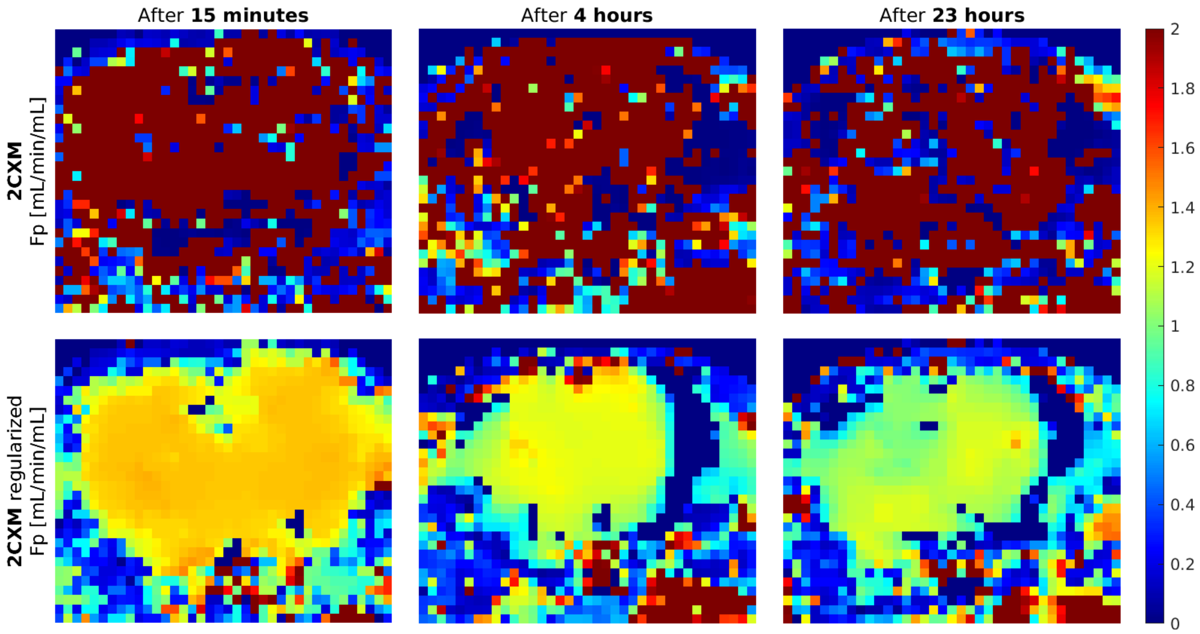

Figure S2: Plasma flow estimates from the voxelwise and regularized 2CXM on real data for the three measurements after sonication. The voxelwise 2CXM led to non-physiological estimates (~100-1000 mL/min/mL), while the regularized 2CXM led to physiological values (~1 mL/min/mL).
